# Atmospheric Deposition of Microplastics in South Central Appalachia in the United States

**DOI:** 10.1021/acsestair.4c00189

**Published:** 2024-12-26

**Authors:** Adam Elnahas, Austin Gray, Jennie Lee, Noora AlAmiri, Nishan Pokhrel, Steve Allen, Hosein Foroutan

**Affiliations:** 1 Department of Civil and Environmental Engineering, Virginia Tech, Blacksburg, Virginia 24061, United States; 2 Department of Biological Science, Virginia Tech, Blacksburg, Virginia 24061, United States; 3 Healthy Earth, 71-75, Shelton Street, Covent Garden, London WC2H 9JQ, U.K.

**Keywords:** airborne microplastics, fiber, passive sampling, Appalachia, Raman spectroscopy

## Abstract

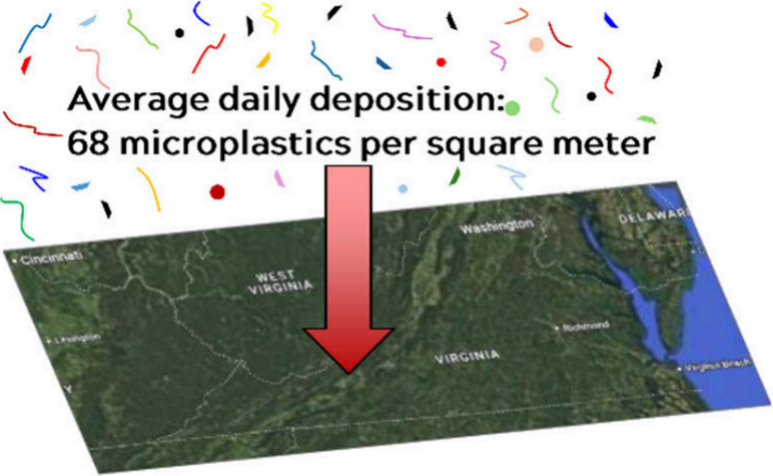

Due to the increased prevalence of plastic pollution globally, atmospheric deposition of microplastics (MPs) is a significant issue that needs to be better understood to identify potential consequences for human health. This study is the first to quantify and characterize atmospheric MP deposition in the Eastern United States. Passive sampling was conducted at two locations within the Eastern United States, specifically in remote South Central Appalachia, from March to September 2023. Each location had five sampling periods, with collections over a 21 day period. Samples were processed to remove biological material, and the presence of MPs was confirmed using Raman spectroscopy to match particles based on polymer similarity. The relative average atmospheric MP deposition in South Central Appalachia was determined to be 68 MPs m^–2^ d^–1^. Most verified MPs were fibers, and the most abundant polymer type identified was poly(ethylene terephthalate) PETE. This study’s average MP deposition rate is qualitatively comparable to rates reported in other studies that employed a similar methodology in a similar landscape. Scaling up our measured deposition rate to all of South Central Appalachia, an area of over 94,000 km^2^ and home to five million people, suggests a yearly MP deposition of approximately 321 metric tonnes. Our study highlights the prevalence of MP deposition in the Eastern United States, providing baseline data for future work to further assess routes of MP introduction.

## Abstract 2

There is currently no research on the atmospheric deposition of microplastics in the Eastern United States. This study quantifies and characterizes atmospheric microplastic deposition at two locations in South Central Appalachia.

## Introduction

Global plastic production is at an all-time high with 460 million tonnes (Mt) of plastic being produced in 2019.^1^ This annual rate of plastic production has risen drastically from 234 Mt of plastics being produced in 2000 and is expected to continue to grow.^1^ The weathering (physical) and degradation (UV, chemical, microbial) of plastics over time allow for the creation of much smaller microplastics (MPs) to be formed. Due to their small size (1 μm - 5 mm), MPs are difficult to contain and can be found in many unique environments such as rivers,^2^ oceans,^3^ food,^4^ and even Antarctic snow.^5^ Over the decades of industrial plastic production and the associated weathering that occurs, the environment is rich with MPs. In fact, studies suggest that MPs are capable of being deposited from the atmosphere in remote locations due to processes such as wind transport and precipitation.^6−8^ MPs deposited from the atmosphere are especially alarming because they can contaminate remote areas once considered pristine. Additionally, people risk inhaling airborne microplastics, and these particles can land on crops, which could then be ingested by humans or wildlife. The additional degradation of MPs leads to the formation of nanoplastics (<1 μm), which become even easier to enter the body.^9^ Microplastics and nanoplastics (MNPs) can pose significant harm to human health, and their presence in the body has even been linked to cases of pulmonary fibrosis, heart attacks, strokes, and death.^10,11^ Because the atmospheric deposition of MPs is highly concerning for public health, there is a strong need to better understand this transport pathway for MPs.

The investigation of atmospheric MP deposition is a field that has grown significantly over the past few years. There have been many studies worldwide that have been researching this in remote locations such as the French Pyrenees,^7^ protected areas in the Western United States,^8^ and Mount Derak, Iran,^6^ as well as urban areas such as Dongguan, China^12^ and London, UK.^13^ Specifically in the United States, the data collected on the atmospheric deposition of MPs are limited to the Western half of the United States. While the quantification of MP deposition is crucial, there was an absence of data on atmospherically deposited MPs in the Eastern half of the United States. Since approximately 59% of Americans live in the Eastern United States,^14,15^ this quantification is necessary to better predict health implications of MP exposure pertaining to the majority of Americans.

Due to the potential for regional variability in this deposition data, this paper sets out to determine if there is atmospheric deposition of MPs in the Eastern United States, specifically in the remote American South Central Appalachian region (South Central Appalachia), and if so, to quantify the deposition rates and determine the characteristics of these MPs.

## Materials and Methods

The details of the methodology followed in this work are presented step-by-step in a tutorial format in the Supporting Information document as well as in Elnahas and Foroutan (2024).^16^ We hope this effort helps with the reproducibility of our results and addresses the current lack of a standard protocol for atmospheric sampling of microplastics, as discussed in several review papers on this topic.^17−19^ Our methodology is briefly described below.

### Sample Collection

**Figure 1:**
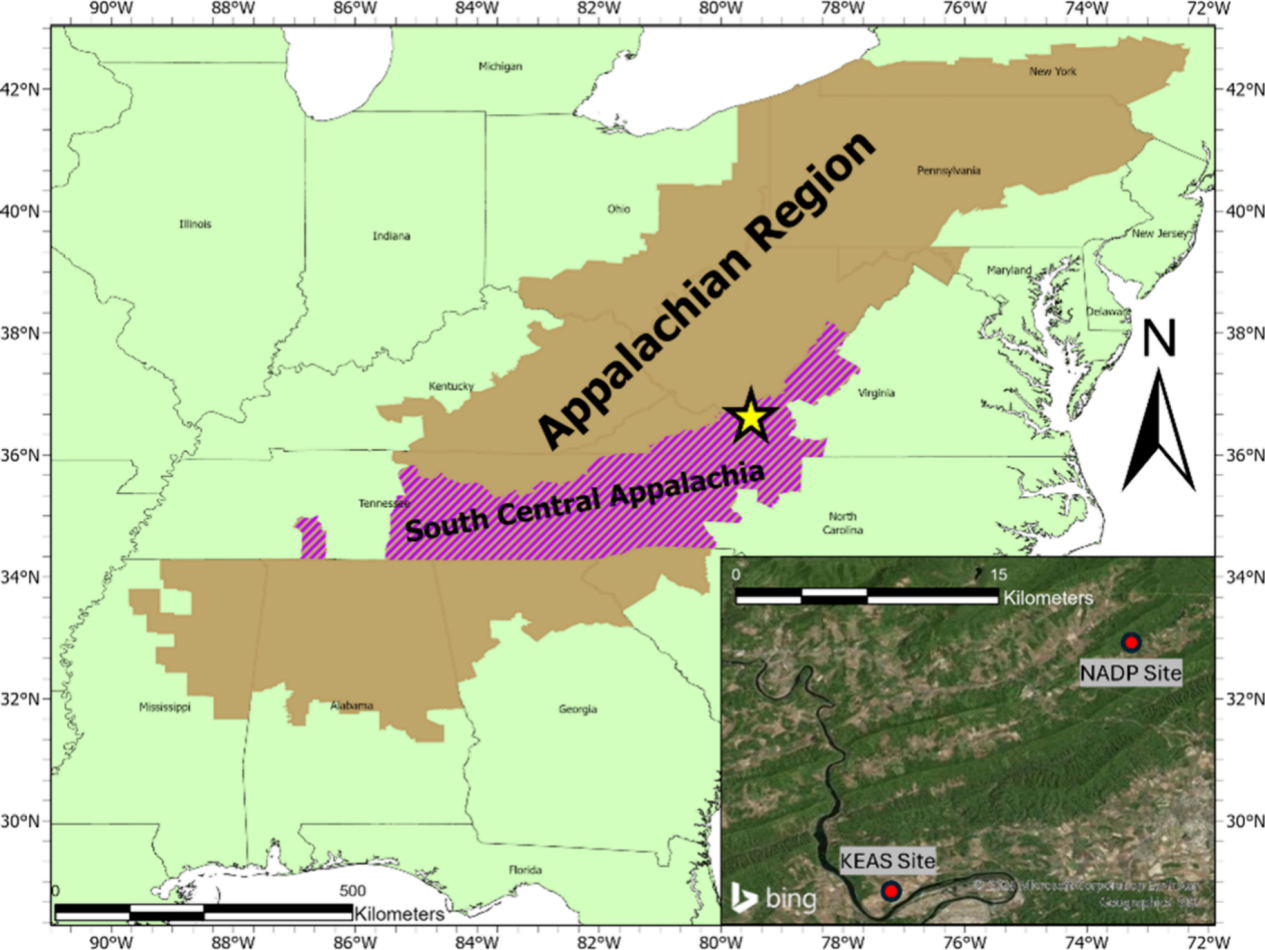
Site map of South Central Appalachia and the greater Appalachian Region sourced from the Appalachian Regional Commission (ARC).^23^ The star represents the inset region that highlights sampling locations. The shapefiles for the regions were sourced from the ARC.^24,25^

Atmospheric deposition of MPs was measured at two remote locations (Figure 1): the National Atmospheric Deposition Program (NADP) Site NTN VA13 and the Kentland Experimental Aerial Systems (KEAS) Site. The NADP Site (37.32°N, 80.46°W) also serves as a Clean Air Status and Trends Network (CASTNET, site VPI120) monitoring site under the United States Environmental Protection Agency. The KEAS Site (37.20°N, 80.58°W) is under the management of Virginia Tech. These sites were selected for sampling because they are private, rural locations with minimal human interaction.^20,21^ Rural areas are defined as having a population density of 10–99 people/km^2^ within a 15 km radius of the site, as per the NADP Site Selection and Installation Manual.^20^ We classified these sites as “rural”, synonymous with our classification of them as “remote locations”. They are expected to be representative of broader conditions in the South Central Appalachia, as both sites are located within this subregion. The subregion, like all Appalachian subregions, is considered relatively homogeneous in terms of topography, demographics, and economics.^22^

Total deposition was collected from the two sites over five 21-day sampling events, each between March 2023 and September 2023. Both locations had the same date ranges for these sampling events. Field blanks were also used to detect any background contamination and quantify it as best as possible. Four field blanks were collected at each sampling location for this study.

Passive atmospheric sampling was conducted using an open beaker, as recommended in previous studies comparing methods for collecting airborne microplastics.^26^ An empty stainless steel beaker (volume: 12 L; base area: 0.043 m^2^) was rinsed twice with acetone and, subsequently, Milli-Q water. The beaker was dried and then immediately covered with tinfoil. This process was repeated for an empty, 1000 mL glass beaker that served as a field blank. The beaker was then secured in place over the duration of the sampling period (SP). The tinfoil was removed from the stainless steel beaker to expose it to the atmosphere, and the researchers immediately left the area. After 21 days, the researchers returned to the locations to collect the stainless steel beakers. The beakers were immediately covered with tinfoil upon returning to the site and taken back to the laboratory. For the field blank samples, the tinfoil was removed from the beaker to expose it to the atmosphere for 1 min before covering it again. To minimize contamination as best as possible, beakers were kept covered and secure in a car when on their way to and from the sites. Additionally, cotton clothing was worn when handling samples and it was crucial that researchers stood downwind when collecting and deploying samples.

### Sample Preparation

Since all field samples contained both wet and dry deposition, excess rainwater was filtered out. The rainwater mixture from the stainless steel beakers was filtered through a 25 mm polycarbonate (PCTE) filter with a pore size of 0.2 μm using a filtration setup consisting of a filter flask, Büchner funnel, and vacuum tube. Any visible remaining contents were gently scraped off of the mixture with a stainless steel spatula. The stainless steel beakers were then rinsed with 96% ethanol and continued to be filtered at least twice more.^7^ The field blanks were rinsed with 96% ethanol and filtered through the filtration setup at least twice since they did not contain wet deposition. The samples underwent organic matter digestion to remove excess organic matter from each of the filters. This process used 30% hydrogen peroxide to rinse each filter.^7^ The filter contents were then placed in a new, clean beaker, covered with tinfoil, and baked for 5–7 days at 55 °C to ensure that the hydrogen peroxide was completely dissolved.^7^ After the samples were baked, the walls of each beaker were rinsed with Milli-Q water at least twice and filtered onto a new PCTE filter using the filtration setup. Any visible remaining contents were gently scraped off of the solution with a stainless steel spatula. The walls of each beaker were then rinsed with 96% ethanol and filtered at least twice. Once the beakers were fully filtered, each finalized filter was then placed onto a microscope slide, secured in place with electrical tape, and placed in a Petri dish.

### Characterization and Quantification

The finalized filters were scanned using a fluorescence microscope (EVOS FL Auto Imaging System, Thermo Fisher Scientific Inc., Waltham, MA) at 10× magnification and a Brightfield setting. Those scans were subsequently inspected using the ImageJ software^27^ to analyze 50% of each filter’s total area for potential MPs.^7^ Potential MPs were visually identified using criteria established by Hidalgo-Ruz et al. in 2012.^28^ Specifically, this was done by ensuring that potential microplastics did not contain any visible cellular or organic structures, were relatively homogeneous in color, and fibers appeared to have a consistent thickness.^28^ Total counts of potential MPs across half of the area of each filter were doubled to estimate the total counts of potential MPs for each filter.

Suspected MPs were visually identified using a stereomicroscope (Leica EZ4, 231 magnification 8–35×). They were characterized based on particle shape (fiber, fragment, or film), color, texture, and other morphological features.^28,29^ Due to a high background interference from using polymeric filters, an issue noted by others such as Brahney et al.,^8^ a subset of particles was isolated and moved to double-sided Scotch tape and analyzed for polymer composition using an XploRA PLUS Confocal Raman Microscope (HORIBA) and processed using LabSpec software (version 6.5).^30−32^ The micro-Raman spectroscopy was conducted utilizing a 532 nm laser with 600 gratings mm^–1^ and a Raman shift range of 100–3,500 cm^–1^. The spectroscopy also used an acquisition time of four s and four accumulations with a maximum laser filter of 25%. After acquiring spectra with Raman spectroscopy, spectra were input to the Open Specy spectra analysis software^33^ and verified to be MPs if they yielded a Pearson’s *R*-value ≥0.7^33,34^ for plastic materials.

The verified proportion of MPs from the subset of total potential MPs was proportionally scaled to estimate the total amount of MPs over the entire area of each filter. Approximately 5% of the total amount of potential MPs on each field sample was analyzed, and approximately 12.5% of the total amount of potential MPs on each field blank was analyzed. These percentages were determined by choosing the largest, reasonable percentage of MPs to analyze given the limitations of resource availability. Since the samples are washed and filtered several times during postprocessing, the final filter is expected to be relatively homogenized, allowing for a smaller portion of the filter to be analyzed. The choice of analyzing 5% of the filter is consistent with similar studies in the literature.^13^ A larger percentage of potential MPs were analyzed on the field blanks because there were typically far fewer potential MPs on them.

## Results and Discussion

### Physicochemical Properties of Deposited MPs

The presence of 35 MPs within field samples was confirmed by using Raman spectroscopy. MPs were identified at each site for each sampling period. The blanks that were collected yielded an average of 8 MPs per sample (7 fragments, 1 fiber, and 0 films). The average count of MPs per field blank was subtracted from the total estimated MPs in each field sample. This subtraction allowed for blank-corrected estimates of the total amount of microplastics on each filter to be determined. After dividing this value by the stainless steel beaker area (0.043 m^2^) and sampling period length (21 days), blank-corrected estimates for the atmospheric deposition of MPs in South Central Appalachia were able to be calculated. The raw data collection used for the verification of potential MPs is presented in the Supporting Information.

**Figure 2:**
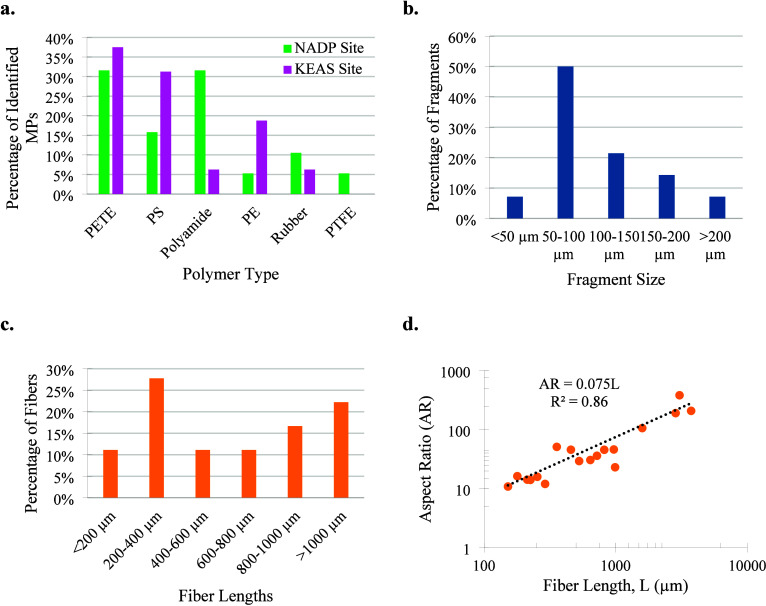
MP Characterization: (a) Polymer type. (b) Fragment size. (c) Fiber length. (d) Fiber aspect ratio.

Out of the 35 confirmed MPs across all field samples, the polymer types break down as follows: polyethylene terephthalate (PETE) 34%, polystyrene (PS) 23%, Polyamide 20%, polyethylene (PE) 11%, rubber 9%, and polytetrafluoroethylene (PTFE) 3%. The polyamide category includes nylon MPs, and the PE category includes PE, high-density polyethylene (HDPE), and PE chlorosulfonated. The breakdown of MP polymer types found at each location is shown in Figure 2a.

PETE consisting of the largest percentage was interesting because, in 2019, PETE consisted of only 7.9% of the total global plastic production.^35^ Furthermore, in 2019, PP made up 19.4% of the total global plastic production,^35^ yet in this study, no PP was verified. There were three potential MPs that had similar spectra to those of PP in Open Specy. However, they were discarded from further analysis due to not having a Pearson’s R-value greater than or equal to 0.7 for their similarity. It is possible that those potential PP MPs, just like other potential MPs that had a weaker Pearson’s *R*-value, represent MPs but were degraded too much, preventing the acquisition of more similar spectra. This has been demonstrated before by Araujo et al.,^36^ where MPs had lower signatures due to degradation and the presence of blended polymers. Some factors that contribute to the degradation of MPs include exposure to UV light, heat, and biodegradation, which cause MPs to continually break down.^36^ This can cause MPs that have been in the environment for a long period of time to have weaker signatures, especially because of the excess natural degradation they undergo. Furthermore, it is possible that there are PP traces in some of the other MPs as well, such as in the polyamides and rubber identified.

The verified MPs had a size range of roughly 39–230 μm for fragments, 150–388 μm for films, and 152–3743 μm for fibers. More than half of all verified MP fragments were less than 100 μm in size. Around 7% of all fragments were smaller than 50 μm, with the smallest observed fragment being 39 μm. Only three of the 35 confirmed MPs were films.

The largest percentage of MP fibers fell between lengths of 200–400 μm, with the second largest percentage consisting of fibers >1000 μm. To further characterize fibers, the ratio of their lengths to widths, or aspect ratio (AR), was determined. Plotting the AR against recorded fiber lengths appeared to convey a linear trend, with an *R*^2^ value of 0.86. Most MP fibers were discovered to have an AR < 50 and a fiber length <1000 μm. Figure 2b–d shows the size distribution of verified fragments and fibers and the trend in the aspect ratio of MP fibers.

The shape breakdown for all confirmed MPs is as follows: fibers 51%, fragments 40%, and films 9%. Both locations had similar percentages of fiber, fragment, and film deposition. It is understandable that the majority of identified MPs in these remote locations are fibers because fibers are capable of traveling longer distances more easily because of their low settling velocities.^37,38^ Since there is no populous urban development near the NADP and KEAS sites to generate large amounts of plastic pollution, it makes sense that most MPs found would need to travel longer distances through the atmosphere to reach the remote sampling locations.

**Figure 3:**
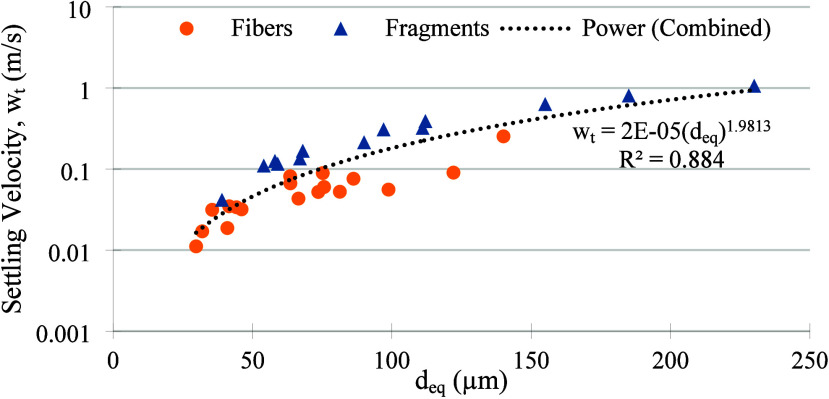
Settling velocities (*w*_t_) for the verified fragment and fiber MPs. The power law relationship between *w*_t_ and *d*_eq_ was fitted through a combination of both types of MPs.

The settling velocity of airborne microplastics (MPs) is an important factor governing their residence time, transport, and fate in the atmosphere. Here, the settling velocities (w_t_) of collected MP fragments and fibers were calculated by using their drag coefficient. The corresponding density based on their polymer type, as defined by our Raman analysis, was used. To account for the Reynolds number and MP shape, we used the drag coefficient scheme developed by Clift and Gauvin^39^ for fragments, and that developed by Bagheri et al.^40^ for fibers. Figure 3 shows the results collectively based on the volume equivalent diameter (d_eq_) of the recorded MPs. In calculating volumes, a cylindrical shape was assumed for fibers and a spherical shape for fragments, following the procedures of Long et al.^41^ and Tatsii et al.^38^

Overall, it is observed that the settling velocity of collected MP fibers is less than that of fragments, even at the same volume equivalent diameter. The majority of fibers had a settling velocity between 0.01 and 0.1 m/s, while most of the fragments had a settling velocity 1 order of magnitude higher, between 0.1 and 1 m/s. Estimating the local spring-summer boundary layer height to be approximately 2 km, the atmospheric lifetime of MP fibers and fragments deposited in the South Central Appalachia due to dry deposition is estimated to be between 5 and 55 h and 0.5 to 5 h, respectively. Altogether, there appears to be a strong power law relationship between the combined *w*_t_ and *d*_eq_ for both fibers and fragments, with an *R*^2^ value of around 0.88.

### MP Deposition Rate

The calculated deposition rates for each sampling period and location are shown in Table 1, and the percent uncertainty for each deposition rate is given in parentheses. The uncertainty values were determined following the methods of Brahney et al.^8^ based upon a percent error calculation for the standard deviation of field blanks in relation to each sample’s blank-corrected deposition rate.

**Table 1:** Deposition Rates for Each Site, with the Percent Uncertainty for Each Value Given in Parentheses

Sampling Period (SP)	Location	Deposition Rate (MPs m^–2^ d^–1^)
1 (3/22/23–4/12/23)	NADP	56 (11%)
	KEAS	37 (17%)
2 (4/27/23–5/18/23)	NADP	57 (11%)
	KEAS	125 (5%)
3 (5/31/23–6/21/23)	NADP	80 (8%)
	KEAS	59 (11%)
4 (7/2/23–7/23/23)	NADP	77 (8%)
	KEAS	35 (18%)
5 (8/19/23–9/9/23)	NADP	100 (6%)
	KEAS	56 (11%)

Across all sampling periods, the NADP location had a mean deposition rate of 74 MPs m^–2^ d^–1^ and a standard deviation of roughly 18.3 MPs m^–2^ d^–1^. The KEAS location had a slightly smaller mean deposition rate of 62 MPs m^–2^ d^–1^, but a standard deviation of around 36.7 MPs m^–2^ d^–1^. For most sampling periods, the NADP location typically had a higher relative deposition rate than the KEAS location. However, in SP2, the KEAS location had a relative deposition rate more than double that of the NADP location.

To quantify the significance of the differences observed in mean deposition rates between the two locations, the Welch two-sample *t* test was conducted. This test was chosen since the NADP and KEAS sites had unequal variances, had deposition independent of each other, and had normally distributed deposition (as determined by a Shapiro Test with *p* > 0.05). The Welch two-sample *t* test maintained a p-value of roughly 0.55 (*p* > 0.05), which allowed to fail to reject that there is no difference in the mean deposition rates for the NADP and KEAS sites. This permits the assumption that the mean MP deposition rate at the NADP Site is the same as the mean MP deposition rate at the KEAS Site, which allows for the greater assumption that this calculated deposition rate is applicable to the entirety of the South Central Appalachian region. Consequently, all collected field samples, despite locations, were averaged to develop an overall mean relative deposition rate for the South Central Appalachia of 68 MPs m^–2^ d^–1^.

In order to further estimate the MP deposition to South Central Appalachia, the average weighted MP density was identified based on the polymer types as roughly 1.2065 g/cm^3^.^42^ The average MP volume was also found to be 1.135 × 10^–7^ cm^3^, and the South Central Appalachian Region area is approximately 36,435 mi^2^.^25^ Based on these values, the yearly MP deposition on the South Central Appalachian region is roughly 321 tonnes. According to the ARC, each Appalachian subregion is relatively homogeneous in regard to its topography, demographics, and economics.^22^ This significant homogeneity allows for the further assumption that there is not much regional variability in atmospheric MP emission and deposition within this subregion, allowing this annual deposition of 321 tonnes to be a reasonable estimate.

If this annual MP deposition estimate were applied to the entire Appalachian Region in the Eastern United States, there would be approximately 1,817 tonnes of atmospheric MP deposition across Appalachia annually. However, because any scaling effort has limitations and this region is far larger and less homogeneous than the subregion of South Central Appalachia, this estimate has a high degree of uncertainty.

### Meteorological Considerations

To better understand the meteorological impacts on atmospheric MP deposition, several local meteorological data fields were analyzed, including the number of rainfall events, average daily precipitation, rainfall event intensity, and wind speeds.^43^ Further details can be found in the Supporting Information. At the NADP Site, the strongest correlation between relative deposition rates and meteorological data was a negative correlation with the average wind speed (r < −0.9, *p* < 0.05). However, at that same site, there was a strong and positive correlation between deposition and the average rainfall event intensity (*r* ≥ 0.6). These relationships suggest that at the NADP Site, there is greater atmospheric MP deposition with lower wind speeds and a large average rainfall event intensity.

When analyzing the KEAS Site, the largest positive correlation was between the atmospheric MP deposition and the number of rainfall events (*r* > 0.7), while the largest negative correlation was between deposition and the maximum rainfall event intensity (*r* < −0.6). This suggests that the raw number of rainfall events is the most influential in contributing to atmospheric MP deposition at this site while an increase in the rainfall intensity could actually lead to a decline in this deposition. This implies that this site receives a greater atmospheric deposition of MPs from lighter, more frequent rainfall events compared to more intense and fewer ones.

### Comparison with Previous Studies

While the lack of a uniform standard allows researchers with the flexibility to investigate the atmospheric deposition of MPs, a true comparison of results is difficult to obtain due to a wide variety of analyses.^19^ This study is expected to be most similar to that of Brahney et al.^8^ since both studies involve the passive sampling of atmospherically deposited MPs within the contiguous United States and maintain other important similarities. A key similarity between this study and Brahney et al.^8^ includes the filtering of samples through a polymeric filter (PES for them, PCTE for this study) that yielded difficulties in identifying in situ due to a higher background signature impacting spectral analysis. Like Brahney, this study also transferred over 200 fibers and particles for spectral analysis.^8^ When determining results to report yearly estimates for regional MP deposition, as done by Brahney et al.,^8^ this study assumed a square shape of particles (fragments and films) with a mean depth of 5 μm to determine the average particle volume and excluded fiber sizes greater than 2,500 μm to determine average fiber volumes. Because many of the procedures are similar between this study and Brahney et al.,^8^ a comparison of results between the two studies does not seem farfetched.

To supplement this comparison to Brahney, Table 2 highlights key comparisons between this study, Brahney et al.,^8^ Allen et al.,^7^ and Abbasi and Turner.^6^ These other studies were chosen for comparison since they also represent deposition rates for atmospherically deposited MPs in remote environments.

**Table 2:** Comparison of Results between This Study and Similar Studies Investigating MP Deposition in Remote Regions Using Passive Sampling^a^

Sampling Location	Deposition Rate (MPs m^–2^ d^–1^)	Size Range (microns)	MP Shape Distribution	Polymer type distribution	MP Verification Method	References
South Central Appalachia (Eastern U.S.)	68	39–3743	Fibers: 51%	PETE: 34%	μRaman	This study
			Fragments: 40%	PS: 23%		
			Films: 9%	Polyamide: 20%		
				PE: 11%		
				Rubber: 9%		
				PTFE: 3%		
American Protected Wilderness (Western U.S.)	132	4–3000	Fibers: 70%	PETE: 22%	FTIR	Brahney et al. (2020)^8^
			Particles: 30%	PS: 11%		
				PE: 9%		
				Other: 58%		
Mount Derak, Iran	12	<100–>1000	Fibers: >95%	PS: 31%	μRaman	Abbasi and Turner (2021)^6^
			Fragments: <5%	PE: 24%		
			Films: 0%	PP: 45%		
French Pyrenees	365	<25–5000	Fibers: 11%	PETE: 2%	μRaman	Allen et al. (2019)^7^
			Fragments: 68%	PS: 41%		
			Films: 21%	PE: 32%		
				Other: 25%		

a Note: The polymer type distribution for Abbasi and Turner (2021) includes data for their joint sampling effort in Shiraz, Iran. These percentages were appropriately scaled to be representative of deposition on Mount Derak since there was only PS, PP, and PE identified at that location.

This study along with Brahney et al.^8^ recorded the largest percentage of MP polymer types to be PETE at 34% and 22%, respectively. However, Allen et al.^7^ instead found a lower percentage of PETE among verified MPs at 2% and a much higher percentage for PS at 41%. This was similarly noted in Abbasi and Turner^6^ since there was no PETE identified at this site, however there was a larger percentage of PS at 31%. This variation is likely due to regional differences in plastic production and waste generation, which could influence the polymer types collected at different locations.

For further comparisons, the Allen study, this study, the Abbasi and Turner study, and the Brahney study conducted field sampling efforts across roughly 5 months, 6 months, 12 months, and 23 months, respectively. All studies were conducted across at least 5 months of field sampling with the Brahney study conducting the most at around 23 months across 11 sampling locations. For reference, this study sampled at two locations; the Abbasi and Turner study sampled at one location on Mount Derak itself (two locations including Shiraz City), and the Allen study sampled at one location within the French Pyrenees.

Furthermore, with regard to laboratory processes, there was much similarity regarding the digestion of organic material. This study, the Allen study, and the Abbasi and Turner study all used hydrogen peroxide to remove excess organic materials from our samples. In contrast, the Brahney study did not conduct organic matter digestion. While using organic matter digestion can certainly be helpful in the visual identification phase for quantifying potential microplastics, it is not necessary to achieve results.

Average MP deposition rates are quite variable, with the Abbasi and Turner study having an average of 12 MP m^–2^ d^–1^ and the Allen study reporting an average of 365 MP m^–2^ d^–1^, while this study and the Brahney study report average deposition values within this range. Due to the large variability within data, it is essential to have a universal MP sampling methodology implemented to better compare worldwide results and better understand trends. (We attempted to partially address this limitation by providing our step-by-step methodology in the Supporting Information, aiming to enhance the reproducibility of our results and facilitate comparisons with future studies).

### Error Mitigation and Limitations

The most significant aspect of error mitigation is likely the analysis of field blanks to determine potential MP contamination. Additionally, lab coats or cotton/linen clothes were worn when working with samples to avoid MP contamination from clothing. When deploying or picking up samples from the NADP and KEAS sites, it was essential to ensure that researchers stood downwind of the samples so as to avoid risking contamination of the field samples. Furthermore, plastic products were avoided as much as possible during the laboratory procedures. Glass products such as beakers, pipettes, Petri dishes, and microscope slides as well as steel products such as a spatula, tweezers, and field samplers were all used to minimize plastic exposure in the lab.

While errors were attempted to be mitigated as best as possible, these results are nevertheless not without error. Since only a subset of the total potential MPs were analyzed for each sample, the deposition rates calculated are only relative values for each sampling period and location. The true deposition rates for these sites could be smaller or larger than these predicted rates; however, the true value would not be possible without completing a full analysis of all potential MPs on all samples.

While the process of isolating potential MPs for Raman analysis was efficient and necessary with the high polymer background from finalized PCTE filters, that removal process is also believed to have restricted the sizes of identified MPs. The smallest recorded MP was a 39 μm fragment, and if the isolation process was not necessary, one could analyze potential MPs directly on their finalized filter. This direct analysis method would be beneficial if using nonpolymeric filters for samples since the background should not cause much interference with the Raman spectroscopy analysis and the spectral acquisition of smaller potential MPs could be performed. We believe that there were MP particles smaller than 39 μm within our samples since there appears to be greater atmospheric deposition of MPs as particle size decreases.^44,45^ However, due to difficulties in analyzing those smaller particles, their presence was unable to be confirmed within our samples. Additionally, when initially developing this study’s methodology, an aluminum oxide Anodisc filter was used; however, its rough surface texture made the visual identification of potential MPs difficult. Because of this, we recommend the use of a different nonpolymeric filter with less surface roughness.

Furthermore, this study did not consider the resuspension of MPs already deposited in the atmosphere at the sampling locations. The process of resuspension would affect the results of this study by decreasing deposition rates at sampling locations due to fewer MPs being deposited directly from the atmosphere. Another error source arises from the final filters for each sample not being perfectly flat for the ImageJ visual counting of potential MPs. This issue, however, would lead to a systematic undercounting of potential MPs which, if corrected, would likely lead to the increase in mean MP deposition rates at each sampling location and a larger estimated yearly MP deposition across all of South Central Appalachia. The most significant limitation for this, and currently all airborne MP studies, is likely the absence of a uniform standard for microplastic sampling and analysis. Without specific guidelines on how the sampling and analysis of atmospherically deposited MPs should be conducted, comparisons between the studies fall short.

### Significance

This study is significant because it is the first estimate of atmospheric MP deposition within the Eastern United States. Furthermore, this study quantifies the estimated MP deposition that falls on South Central Appalachia, home to more than five million people,^23^ every year as over 300 tonnes, which is an incredible amount of atmospheric deposition. The mean atmospheric MP deposition rate reported here, i.e., 68 MPs m^–2^ d^–1^, is believed to be a reasonable value carefully calculated based on 10 samples within a period of 6 months in 2023, accounted for error mitigation such as the inclusion of field blanks, and fell within ranges of results published on atmospheric MP deposition. Due to limitations identifying MPs under 39 μm, there is likely a larger deposition rate of MPs in this region than 68 MPs m^–2^ d^–1^. While there may be more MPs deposited, the yearly deposition of over 300 tonnes should still be roughly the same due to those smaller MPs contributing much less to the overall mass being deposited. Finally, in an attempt to establish a standardized approach for collecting and analyzing atmospheric MP fallout, we included a step-by-step tutorial outlining our methodology in the Supporting Information. This can be utilized by other researchers in the field and facilitates intercomparisons between studies.

### Future Work

In order to better quantify and characterize MP deposition within South Central Appalachia, more locations within the region need to have sampling performed. In order to identify smaller MPs within samples, nonpolymeric filters should be utilized to allow for the better in situ analysis of potential MPs using Raman spectroscopy. Additionally, both wet and dry deposition should be individually investigated within South Central Appalachia to clarify the impact of meteorological phenomena on atmospheric MP deposition within the Eastern United States. The majority of the United States population lives in its eastern part, especially in urban areas, so atmospheric MP deposition in these more populous environments needs to be quantified and characterized. Microplastics can pose an immediate risk to human health,^10,11^ and so their routes for exposure need to be further understood. However, before researching atmospheric MP deposition in urban areas in the Eastern United States or taking more passive deposition samples at more sampling locations in South Central Appalachia, a standard methodology needs to be implemented to allow for better comparisons between studies.

## Supporting Information Available

Additional experimental details, materials, and methods (PDF)

## Abbreviations

ARC: Appalachian Regional Commission
NADP: National Atmospheric Deposition Program
KEAS: Kentland Experimental Aerial Systems
CASTNET: Clean Air Status and Trends Network
SP: sampling period
PCTE: polycarbonate track etch
PETE: polyethylene terephthalate
PS: polystyrene
PE: polyethylene
PTFE: polytetrafluoroethylene
HDPE: high density polyethylene
AR: aspect ratio
NTN: National Trends Network
PP: polypropylene
PES: poly(ether sulfone)
