# Supplementary material for: Atmospheric Deposition of Microplastics in South Central Appalachia in the United States
Source: ACS EST Air. 2024 Dec 26;2(1):64–72. doi: 10.1021/acsestair.4c00189 (PMC11730859; doi:10.1021/acsestair.4c00189)
Supplement: Supplementary file 1 — ea4c00189_si_001.pdf [file ea4c00189_si_001.pdf]

SUPPORTING INFORMATION

Atmospheric Deposition of Microplastics in South Central Appalachia in the United States

Adam Elnahas[1], Austin Gray[2], Jennie Lee[1], Noora AlAmiri[1], Nishan Pokhrel[1], Steve Allen[3], Hosein Foroutan[1,*]

[1] Department of Civil and Environmental Engineering, Virginia Tech, Blacksburg, Virginia 24061, USA

[2] Department of Biological Sciences, Virginia Tech, Blacksburg, Virginia 24061, USA

[3] Healthy Earth, 71-75, Shelton Street, Covent Garden, London WC2H 9JQ, UK

* Corresponding Author: Hosein Foroutan, hosein@vt.edu

This supporting information document contains the following information:

- Meteorological data from the Virginia Tech weather station
- Raw data used for MP verification
- Additional figure for comparison between previous airborne MP studies
- ECDF plots comparing deposition size distributions for studies mentioned in Table 2
- Detailed Materials and Methods

Number of pages: 20

Number of figures: 3

Number of tables: 1

Meteorological data from the Virginia Tech Weather Station

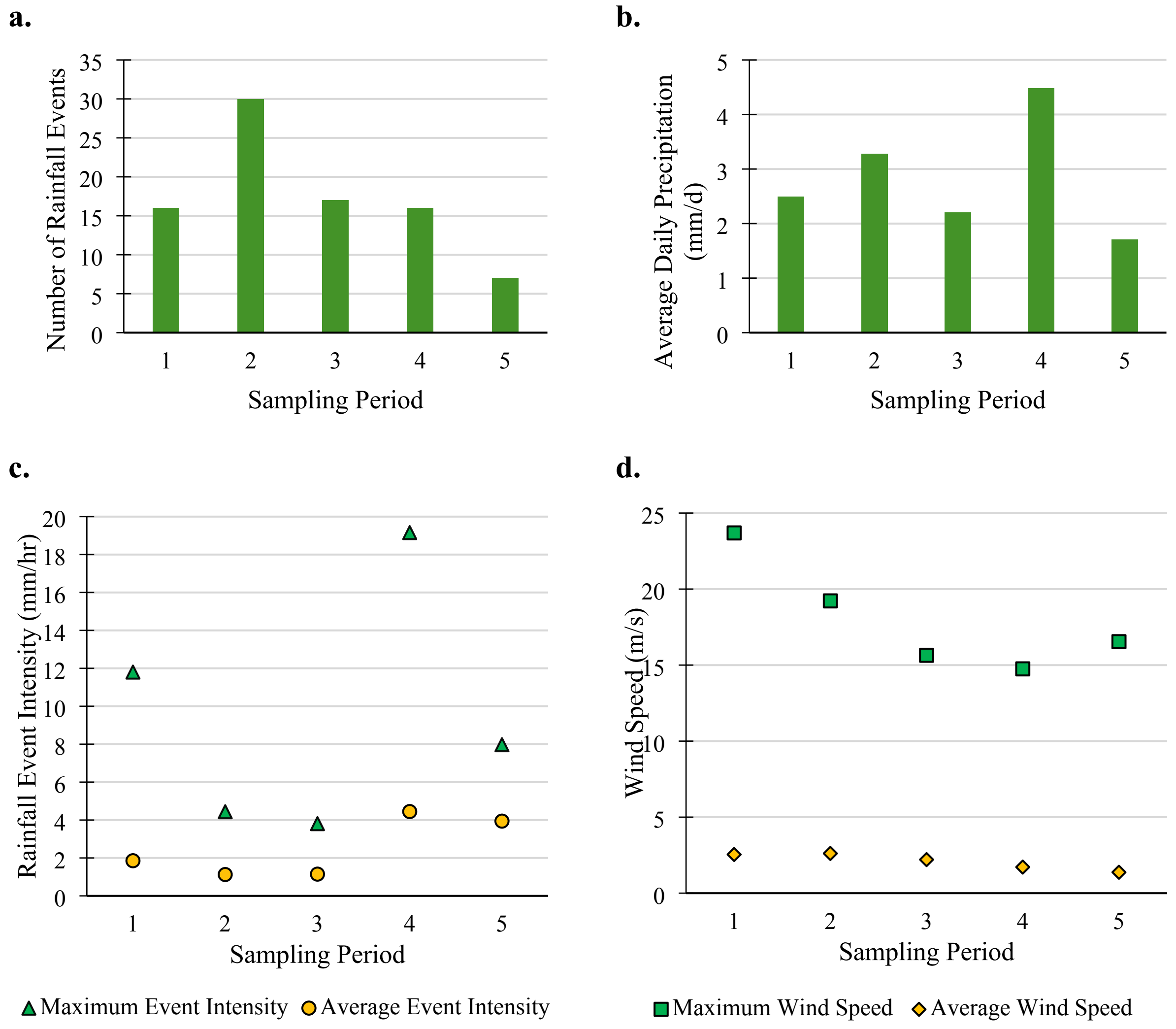

Figure S1. Meteorological data. **a,** Quantification of rainfall events during SPs. Rainfall events are defined as continuous precipitation events with no more than 30 minutes between lull periods. **b,** Average daily precipitation during SPs. **c,** Rainfall event intensities during SPs. **d,** Wind speeds during SPs. Meteorological data was taken from WeatherSTEM for Montgomery County, VA using the Virginia Tech station [1].

Table S1. Raw data used for MP verification

Sampling Period	Location	Blank ?	ID #:	Open Specy Polymer Characterization	Pearson's R	Shape	Size (micron)	Color
1	NADP	no	1	**rubber**	**0.83**	fragment	111	black
1	NADP	no	2	N/A	N/A	fragment	N/A	gray
1	NADP	no	3	**pvc**	**0.66**	fragment	163	yellow
1	NADP	no	4	N/A	N/A	fragment	N/A	black
1	NADP	no	5	N/A	N/A	fragment	N/A	black
1	NADP	no	6	N/A	N/A	fragment	N/A	gray
1	NADP	no	7	N/A	N/A	fiber	N/A	black
1	NADP	no	8	**nylon_6_9**	**0.79**	fiber	291	black
1	NADP	no	9	N/A	N/A	fragment	N/A	black
1	NADP	no	10	N/A	N/A	fragment	N/A	red
1	NADP	no	11	N/A	N/A	fragment	N/A	black
1	NADP	no	12	N/A	N/A	fragment	N/A	black
1	NADP	no	13	**polycarbonate**	**0.52**	fiber	1754	gray
1	NADP	no	14	N/A	N/A	fragment	N/A	black/gray
1	NADP	no	15	N/A	N/A	fragment	N/A	red
1	NADP	no	16	N/A	N/A	fragment	N/A	black
1	NADP	no	17	N/A	N/A	fiber	N/A	black
1	NADP	no	18	**polystyrene**	**0.82**	fiber	179	gray
1	KEAS	no	1	**polyamide**	**0.88**	fiber	990	clear
1	KEAS	no	2	N/A	N/A	fragment	N/A	black
1	KEAS	no	3	**ps**	**0.68**	fragment	33	black
1	KEAS	no	4	**nylon_6_9**	**0.63**	fiber	349	clear
1	KEAS	no	5	**polybuthylene_terephthalate**	**0.52**	fragment	46	black
1	KEAS	no	6	**pete**	**0.64**	fragment	141	clear
1	KEAS	no	7	**pete**	**0.71**	fiber	642	clear
1	KEAS	no	8	**pp**	**0.61**	fiber	76	black
1	KEAS	no	9	**pete**	**0.62**	fragment	33	orange
1	KEAS	no	10	N/A	N/A	fragment	N/A	black
1	KEAS	no	11	N/A	N/A	fiber	N/A	clear
1	KEAS	no	12	N/A	N/A	fragment	N/A	white
1	KEAS	no	13	**pete**	**0.55**	fragment	74	black
1	KEAS	no	14	**nylon 6 (3) t**	**0.66**	fragment	62	black
2	NADP	no	1	**pe**	**0.65**	fragment	86	white
2	NADP	no	2	N/A	N/A	fiber	N/A	clear
2	NADP	no	3	**pe**	**0.69**	fragment	102	clear
2	NADP	no	4	N/A	N/A	fiber	N/A	clear
2	NADP	no	5	**pete**	**0.64**	fiber	221	orange

2	NADP	no	6	**ps**	**0.9**	fiber	968	clear
2	NADP	no	7	**epdm rubber**	**0.7**	fragment	67	clear
2	NADP	no	8	N/A	N/A	fragment	N/A	clear
2	NADP	no	9	**pp isotactic**	**0.64**	fiber	1709	blue/white
2	NADP	no	10	N/A	N/A	fragment	N/A	cream
2	NADP	no	11	**pvc**	**0.61**	fiber	516	clear
2	NADP	no	12	N/A	N/A	fragment	N/A	red
2	NADP	no	13	N/A	N/A	fragment	N/A	clear
2	NADP	no	14	**isobutylen-isopropen-rubber**	**0.5**	fragment	95	black
2	NADP	no	15	**polyamide**	**0.75**	fiber	357	clear
2	NADP	no	16	N/A	N/A	fiber	N/A	green
2	KEAS	no	1	N/A	N/A	fiber	N/A	black
2	KEAS	no	2	**polystyrene**	**0.56**	fiber	624	black
2	KEAS	no	3	N/A	N/A	fragment	N/A	black
2	KEAS	no	4	**polyethylene**	**0.69**	fiber	113	black
2	KEAS	no	5	N/A	N/A	fiber	N/A	gray
2	KEAS	no	6	N/A	N/A	fragment	N/A	black
2	KEAS	no	7	N/A	N/A	fragment	N/A	gray
2	KEAS	no	8	**polystyrene**	**0.8**	fiber	2854	black
2	KEAS	no	9	**pete**	**0.86**	fragment	230	black
2	KEAS	no	10	**rubber**	**0.76**	fiber	1592	black
2	KEAS	no	11	**polystyrene**	**0.91**	fiber	225	gray
2	KEAS	no	12	**polystyrene**	**0.9**	film	227	clear
2	KEAS	no	13	N/A	N/A	fiber	N/A	white
2	KEAS	no	14	**polystyrene**	**0.83**	fragment	90	clear
3	NADP	no	1	**polyethylene**	**0.67**	fiber	737	black
3	NADP	no	2	**pete**	**0.79**	fragment	112	black
3	NADP	no	3	**cellulose acetate**	**0.57**	fragment	80	red
3	NADP	no	4	N/A	N/A	fragment	N/A	black
3	NADP	no	5	N/A	N/A	fragment	N/A	black
3	NADP	no	6	**pete**	**0.85**	fragment	155	clear
3	NADP	no	7	N/A	N/A	fragment	N/A	red
3	NADP	no	8	**polyamide**	**0.88**	fiber	213	red
3	NADP	no	9	N/A	N/A	fiber	N/A	black
3	NADP	no	10	**polyamide**	**0.88**	film	150	clear
3	NADP	no	11	N/A	N/A	fragment	N/A	white
3	NADP	no	12	N/A	N/A	fragment	N/A	black
3	NADP	no	13	N/A	N/A	film	N/A	clear
3	NADP	no	14	**polyamide_6**	**0.58**	fiber	90	black

3	KEAS	no	1	**nylon**	**0.67**	fiber	286	red
3	KEAS	no	2	**pete**	**0.96**	fragment	58	clear
3	KEAS	no	3	**ps**	**0.91**	fiber	457	clear
3	KEAS	no	4	**pete**	**0.84**	fragment	97	clear
3	KEAS	no	5	N/A	N/A	fiber	N/A	clear
3	KEAS	no	6	N/A	N/A	fibers	N/A	yellow
3	KEAS	no	7	**pe**	**0.65**	fragment	58	clear
4	NADP	no	1	N/A	N/A	fragment	N/A	clear
4	NADP	no	2	**ptfe**	**0.71**	fiber	3056	clear
4	NADP	no	3	**polycarbonate**	**0.67**	fiber	252	black
4	NADP	no	4	**nylon**	**0.88**	fragment	59	white
4	NADP	no	5	**pete**	**0.93**	fiber	152	red
4	NADP	no	6	**pete**	**0.52**	film	88	clear
4	NADP	no	7	**pete**	**0.78**	fragment	54	white
4	NADP	no	8	N/A	N/A	film	N/A	clear
4	NADP	no	9	N/A	N/A	fragment	N/A	black
4	NADP	no	10	N/A	N/A	fragment	N/A	clear
4	NADP	no	11	**pete**	**0.6**	fragment	31	black
4	KEAS	no	1	**rubber**	**0.66**	fragment	44	orange
4	KEAS	no	2	N/A	N/A	fragment	N/A	black
4	KEAS	no	3	N/A	N/A	fiber	N/A	black
4	KEAS	no	4	N/A	N/A	fiber	N/A	black
4	KEAS	no	5	N/A	N/A	fragment	N/A	black
4	KEAS	no	6	N/A	N/A	fragment	N/A	black
4	KEAS	no	7	N/A	N/A	fragment	N/A	black
4	KEAS	no	8	**ldpe**	**0.59**	fiber	1801	white
4	KEAS	no	9	N/A	N/A	fragment	N/A	black
4	KEAS	no	10	**pete**	**0.95**	fragment	185	yellow
4	KEAS	no	11	N/A	N/A	fragment	N/A	black
4	KEAS	no	12	**pa6/pa66**	**0.54**	fragment	95	black
4	KEAS	no	13	N/A	N/A	fiber-short	N/A	black
4	KEAS	no	14	**nylon 6 (3) t**	**0.62**	fragment	102	black
4	KEAS	no	15	**rubber**	**0.66**	fragment	47	black
4	KEAS	no	16	N/A	N/A	fragment	N/A	black
4	KEAS	no	17	**polyethylene**	**0.7**	fragment	39	white/cream
4	KEAS	no	18	N/A	N/A	fragment	N/A	white
5	NADP	no	1	**pe**	**0.66**	fiber	941	black
5	NADP	no	2	**polystyrene**	**0.91**	film	388	clear
5	NADP	no	3	**polyamide**	**0.82**	fiber	3743	white
5	NADP	no	4	**polyamide**	**0.59**	fragment	147	black

5	NADP	no	5	**pete**	**0.61**	fragment	50	white
5	NADP	no	6	N/A	N/A	fragment	N/A	black
5	NADP	no	7	N/A	N/A	film	N/A	clear
5	NADP	no	8	N/A	N/A	fragment	N/A	maroon
5	NADP	no	9	N/A	N/A	fiber	N/A	black
5	NADP	no	10	**pete**	**0.59**	fragment	43	black
5	NADP	no	11	N/A	N/A	fragment	N/A	clear
5	NADP	no	12	**pete**	**0.86**	fragment	112	black
5	NADP	no	13	N/A	N/A	fragment	N/A	black
5	NADP	no	14	N/A	N/A	fragment	N/A	black
5	NADP	no	15	N/A	N/A	fiber	N/A	white
5	NADP	no	16	**poly(vinyl_acetate)**	**0.68**	fiber	590	clear
5	NADP	no	17	N/A	N/A	fragment	N/A	black
5	NADP	no	18	**pp**	**0.62**	fragment	90	clear
5	NADP	no	19	N/A	N/A	fragment	N/A	black
5	NADP	no	20	**polyethylene chlorosulfonated**	**0.73**	fiber	819	red
5	NADP	no	21	**pcl**	**0.55**	fragment	54	clear
5	NADP	no	22	N/A	N/A	fragment	N/A	clear
5	NADP	no	23	N/A	N/A	fragment	N/A	clear
5	NADP	no	24	**pete**	**0.96**	fiber	528	white
5	NADP	no	25	N/A	N/A	fragment	N/A	gray
5	NADP	no	26	**isobutylen-isopropen-rubber**	**0.68**	fragment	68	black
5	KEAS	no	1	**pe**	**0.57**	fibers	>1000, too clustered	white
5	KEAS	no	2	**hdpe**	**0.74**	fiber	721	black
5	KEAS	no	3	N/A	N/A	fragment	N/A	clear
5	KEAS	no	4	**hdpe**	**0.7**	fiber	254	clear
5	KEAS	no	5	**pete**	**0.74**	fragment	68	cream
5	KEAS	no	6	N/A	N/A	fragment	N/A	white
5	KEAS	no	7	N/A	N/A	fiber	N/A	white
5	KEAS	no	8	N/A	N/A	fragment	N/A	black
5	KEAS	no	9	N/A	N/A	film	N/A	clear
5	KEAS	no	10	N/A	N/A	fragment	N/A	clear
5	KEAS	no	11	N/A	N/A	fragment	N/A	black
5	KEAS	no	12	N/A	N/A	fragment	N/A	black
5	KEAS	no	13	N/A	N/A	fragment	N/A	clear
5	KEAS	no	14	N/A	N/A	fragment	N/A	clear
5	KEAS	no	15	N/A	N/A	fiber	N/A	white
5	KEAS	no	16	N/A	N/A	fragment	N/A	orange

2	NADP	YES	1	N/A	N/A	fragment	N/A	clear
2	NADP	YES	2	**nylon**	**0.88**	fiber	821	clear
2	NADP	YES	3	N/A	N/A	fragment	N/A	yellow
2	NADP	YES	4	N/A	N/A	fiber	N/A	white
2	NADP	YES	5	N/A	N/A	fragment	N/A	black
2	KEAS	YES	1	**pete**	**0.65**	fiber	1414	blue
2	KEAS	YES	2	N/A	N/A	fragment	N/A	brown
2	KEAS	YES	3	N/A	N/A	fragment	N/A	black
2	KEAS	YES	4	N/A	N/A	fiber	N/A	white
2	KEAS	YES	5	N/A	N/A	fiber	N/A	black
2	KEAS	YES	6	**pp**	**0.6**	film	67	white
3	NADP	YES	1	**nylon**	**0.88**	fragment	20	black
3	NADP	YES	2	N/A	N/A	fiber	N/A	white
3	NADP	YES	3	N/A	N/A	fragment	N/A	white
3	NADP	YES	4	N/A	N/A	fiber	N/A	white
3	NADP	YES	5	N/A	N/A	fragment	N/A	white
3	NADP	YES	6	N/A	N/A	film	N/A	clear
3	NADP	YES	7	N/A	N/A	fragment	N/A	black
3	KEAS	YES	1	**polyamide_6**	**0.61**	fiber	489	black
3	KEAS	YES	2	N/A	N/A	fragment	N/A	black
3	KEAS	YES	3	**pete**	**0.68**	fiber	529	clear
3	KEAS	YES	4	N/A	N/A	fiber	N/A	clear
3	KEAS	YES	5	N/A	N/A	fragment	N/A	clear
3	KEAS	YES	6	N/A	N/A	fiber	N/A	clear
3	KEAS	YES	7	**nylon**	**0.88**	fragment	18	black
3	KEAS	YES	8	**pete**	**0.52**	fiber	740	clear
3	KEAS	YES	9	N/A	N/A	fragment	N/A	gray
3	KEAS	YES	10	N/A	N/A	fragment	N/A	clear
4	NADP	YES	1	**acrylonitrile butadiene styrene (other plastic)**	**0.62**	fragment	130	black
4	NADP	YES	2	N/A	N/A	fiber	N/A	white
4	NADP	YES	3	N/A	N/A	fiber	N/A	white
4	NADP	YES	4	N/A	N/A	fragment	N/A	clear
4	NADP	YES	5	N/A	N/A	fiber	N/A	clear
4	NADP	YES	6	N/A	N/A	fiber	N/A	white
4	NADP	YES	7	**isobutylen-isopropen-rubber**	**0.6**	fragment	90	clear
4	NADP	YES	8	**nylon**	**0.88**	fragment	25	gray
4	NADP	YES	9	**pete**	**0.64**	fragment	81	clear
4	NADP	YES	10	**pete**	**0.6**	fragment	55	clear

4	KEAS	YES	1	**pete**	**0.64**	fiber	1162	white
4	KEAS	YES	2	N/A	N/A	fragment	N/A	red
4	KEAS	YES	3	**ptfe**	**0.73**	fragment	75	white
4	KEAS	YES	4	N/A	N/A	fiber	N/A	white
4	KEAS	YES	5	**ps**	**0.91**	fragment	139	clear
5	NADP	YES	1	**nylon**	**0.88**	fragment	65	white
5	NADP	YES	2	**pete**	**0.63**	fiber	811	clear
5	NADP	YES	3	N/A	N/A	fragment	N/A	black
5	NADP	YES	4	N/A	N/A	fiber	N/A	white
5	KEAS	YES	1	**pete**	**0.51**	fragment	99	white
5	KEAS	YES	2	**ps**	**0.57**	fragment	94	clear

Notes:

1. Yellow highlighted values represent % Spectral Similarity between 50%-70% (0.5<Pearson's R<0.7)

2. Green highlighted values represent % Spectral Similarity greater than or equal to 70% (Pearson's R>=0.7)

Additional airborne MP comparison

a.

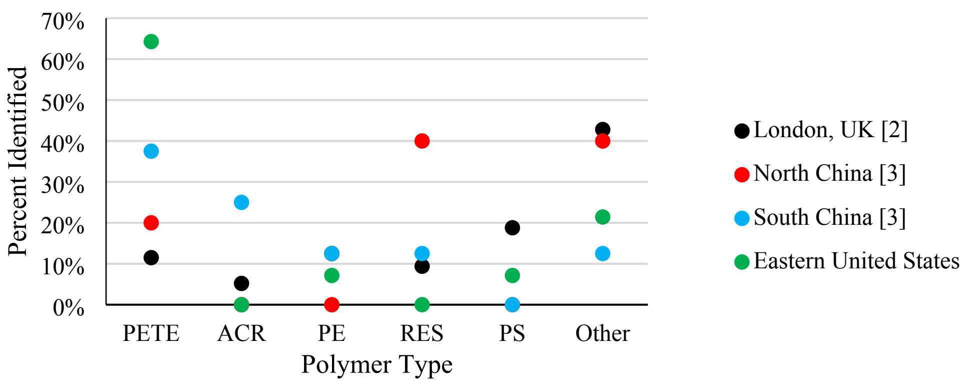

b.

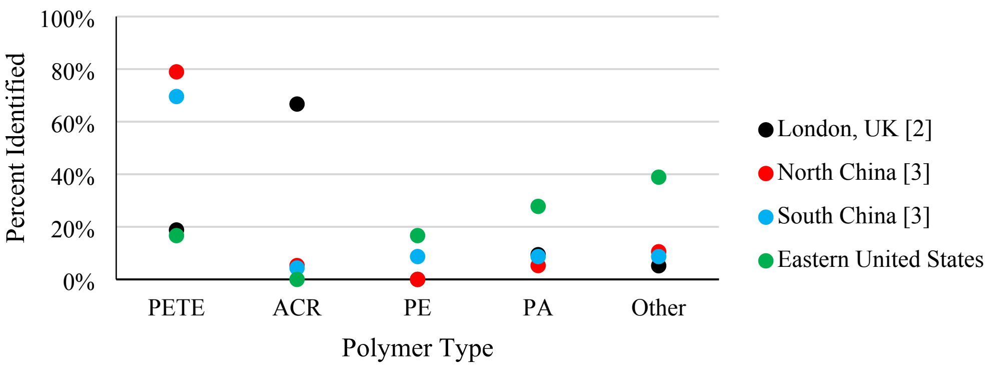

c.

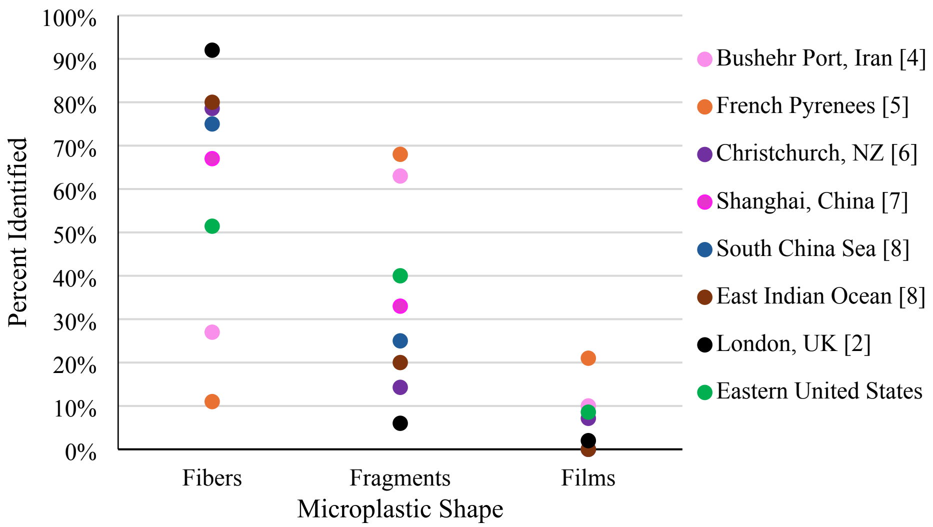

d.

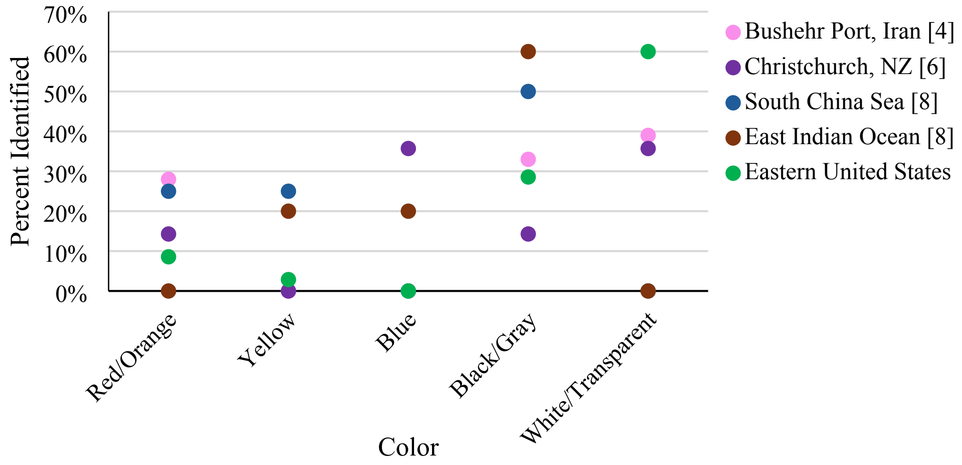

Figure S2. Comparisons of airborne MP data with additional studies around the world. **a,** Fragment MP polymer types across studies. PETE includes polyester. Acrylics (ACR) includes polyacrylonitrile. Resins (RES) includes alkyd resins. **b,** Fiber MP polymer types across studies. Polyamides (PA) includes nylon. **c,** MP shape variations among studies. **d,** MP color variations among studies. White/Transparent category includes "cream" color. Brackets in the legend indicate the reference for each data entry. "Eastern United States" refers to this study.

The verified fragments in this study consisted mainly of PETE, as shown in Figure S2a. In contrast to other studies, this study did not detect any resin fragments. This study also did not detect any acrylic fragments, a similar case to North China. This study did find percentages for PE, PS, and the "Other" category for fragments between the data from the other included studies.

As shown in Figure S2b for MP fibers, this study identified a similar PETE fiber percentage to London, yet quite a lower percentage when compared to North and South China. Conversely, acrylic fiber percentages were similar between this study and North and South China, yet much

lower than the reported percentage in London. This study also recorded larger percentages of MP fibers for PE, PA, and the “Other” category when compared to the other studies included here.

This study yielded a similar trend to various other studies on airborne MPs as noted in Figure S2c with larger percentages of fibers, then fragments, and lastly, films. This trend was consistent for all studies included except for the locations of the French Pyrenees and Bushehr Port, Iran.

In regard to the colors of MPs identified, this study had the largest percentage of verified MPs being white or transparent, as shown in Figure S2d. This high amount of white or transparent MPs being identified could be because of potential bleaching from the use of hydrogen peroxide for the organic matter digestion process. However, this study also recorded percentages of verified MPs being Red/Orange, Yellow, and Black/Gray to be between the percentages of the other studies included. Similarly to Bushehr Port, Iran and the South China Sea, this location did not verify the presence of any blue MPs from samples.

ECDF plots comparing size distributions of MPs from Table 2

a.

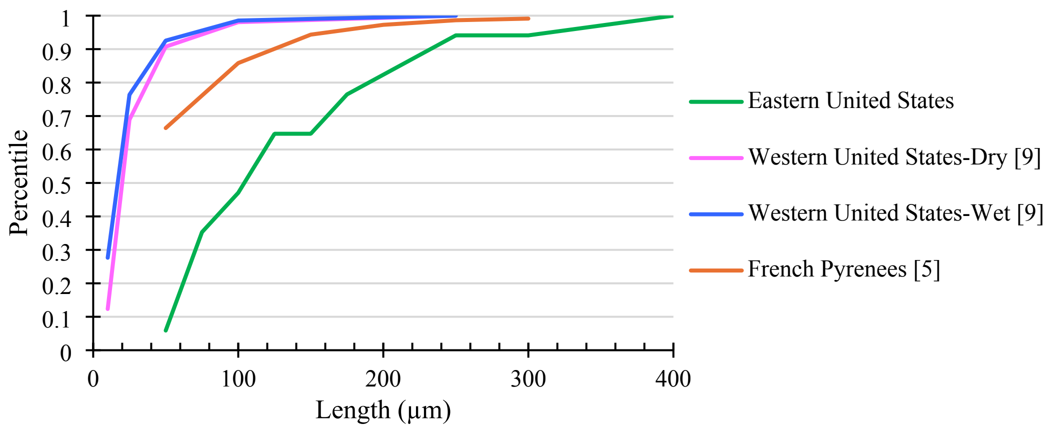

b.

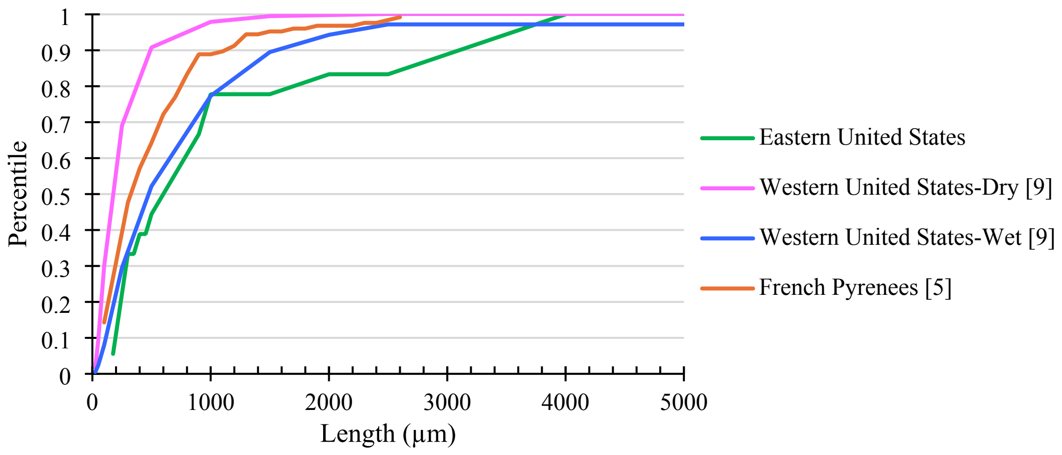

Figure S3. ECDF plots for comparing the size distributions of atmospherically deposited MPs with the other studies from Table 2. **a,** ECDF plot for particle (fragments and films) size distributions. **b,** ECDF plot for fiber size distributions. Brackets in the legend indicate the reference for each data entry.

Additional differences in size distributions of verified MPs for studies within Table 2 are shown as ECDF plots above in Figure S3. The Abbasi and Turner study for Mount Derak [10] was excluded from these ECDF plots due to more limited data availability on verified MP sizes. As shown in Figure S3a., there are quite visible differences in recorded sizes for MP particles within the studies included. However, the Western United States had very similar ECDF plots for both its wet and dry deposition with around 60% of all verified MP particles as being less than ~20 μm in length. The French Pyrenees had around 66% of all their MP particles less than 50 μm in length, with most of their fragments specifically (53%) being less than 25 μm in size. This study for the Eastern United States tended to yield larger size distributions as shown in the ECDF plot, likely due to the aforementioned limitations in verifying MPs smaller than 39 μm.

The ECDF plot for MP fiber sizes shown in Figure S3b. shows more detailed data for smaller percentiles. This allows for an enhanced comparison of the characteristics of fibers across these studies, even though there are no two very similar plots like in Figure S3a. For example, we can see that at the same percentile of 0.2, each ECDF plot had 20% of its MP fibers fall below quite different sizes. At that same threshold, the Western United States dry deposition recorded sizes < ~100 μm, the French Pyrenees recorded sizes < ~150 μm, the Western United States wet deposition recorded sizes < ~200 μm, and the Eastern United States recorded sizes < ~250 μm. These differences in recorded sizes of fibers throughout studies are even more prominent at larger percentiles as well.

Step-by-step methodology for passively investigating the atmospheric deposition of microplastics in South Central Appalachia

How to Cite:

Elnahas, A. & Foroutan, H. (2024). Atmospheric Deposition of Microplastics in South Central Appalachia in the United States - Methodology. University Libraries, Virginia Tech. Dataset. https://doi.org/10.7294/26364370.v1

Site Preparation:

1. Choose a remote location away from much human development and activity.
2. Place a cinderblock vertically on a flat surface at the site.
3. Place two small poles on either side of the cinderblock. Make sure they are placed a few inches into the ground to ensure they are stable.

Sampler and Field Blank Preparation:

1. Rinse a stainless steel beaker (~~V~~ = 12 L) with **acetone****, ACS reagent twice.
2. Rinse the stainless steel beaker twice with Milli-Q water.
3. Dry the stainless steel beaker using the air valve in a fume hood.
4. Immediately cover the dry stainless steel beaker with tinfoil.
5. Repeat steps 1-4 for a 1000 mL glass beaker that will serve as the field blank.

****RECOMMENDATION:** We recommend filtering acetone and any reagent prior to use to reduce background contamination of MPs.

Sampler Deployment:

1. Place the stainless steel beaker on top of the cinderblock at the site.
2. Wrap a wire around the stainless steel beaker to secure it in place with the two adjacent poles.
3. Remove the tinfoil to uncover the FIELD BLANK.
    a. Allow the field blank to be open to the atmosphere for 1 minute.
4. Cover the field blank with tinfoil again.
5. Remove the tinfoil from the stainless steel beaker.
6. Immediately leave the area.
7. Drop off the field blank at the lab.
8. After three weeks (21 days), return to the sampling site.

9. Immediately cover the stainless steel beaker with tinfoil.
10. Drop off the stainless steel beaker at the lab.

Notes: When deploying or picking up samples from the site, it is essential to stand downwind of the samples to avoid the risk of contaminating samples.

Filtration:

If filtering a stainless steel beaker:

1. Place a Büchner funnel rubber stopper on top of a filtering flask.
2. Place a 25 mm Büchner funnel on top of the rubber stopper.
3. Use a vacuum tube to connect the filtering flask to a vacuum source.
4. Place a 25 mm, 0.2 μm pore PCTE filter on top of the Büchner funnel setup.
5. Remove the tinfoil covering the stainless steel beaker.
6. Pour some of the contents into a 1000 mL glass beaker.
7. Cover the stainless steel beaker with tinfoil again.
8. Turn on the vacuum.
9. Slowly pour the contents from the 1000 mL beaker into the filtration setup.
    a. This process can be slow, so cover the 1000 mL beaker with tinfoil to limit indoor MP deposition within the lab.
    b. Pour a little bit incrementally until the 1000 mL beaker is empty.
    c. Turn off the vacuum.
10. Repeat steps 5-9 until the stainless steel container is empty.
11. Use a stainless steel spatula to scrape off any visible debris within the stainless steel beaker.
12. Rinse the stainless steel beaker with 96% ethanol at least twice.
13. Repeat steps 5-9 until the stainless steel container is empty.
14. Rinse the 1000 mL beaker with 96% ethanol at least twice.
15. Turn on the vacuum.
16. Repeat step 9.
17. Use stainless steel tweezers to gently place the filter into a clean, glass beaker.
18. Cover the beaker with tinfoil.

If filtering a glass field blank:

1. Place a Büchner funnel rubber stopper on top of a filtering flask.
2. Place a 25 mm Büchner funnel on top of the rubber stopper.
3. Use a vacuum tube to connect the filtering flask to a vacuum source.
4. Place a 25 mm PCTE filter on top of the Büchner funnel setup.
5. Remove the tinfoil covering the glass field blank.
6. Turn on the vacuum.
7. Slowly pour the contents from the glass field blank into the filtration setup.
    a. Pour a little bit incrementally until the field blank is empty.

    b. Turn off the vacuum.
8. Use a stainless steel spatula to scrape off any visible debris within the glass field blank.
9. Rinse the field blank with 96% ethanol at least twice.
10. Turn on the vacuum.
11. Repeat step 7.
12. Use stainless steel tweezers to gently place the filter into a clean, glass beaker.
13. Cover the beaker with tinfoil.

Notes:

- If you suspect that the filter is getting clogged and is filtering very slowly, turn off the vacuum. Then, gently lift the Büchner funnel to expose the inside of the filtering flask to the atmosphere. Gently lay it back down on top of the rubber stopper and turn on the vacuum again.
- Make sure to dump waste from the filtering flask once it gets too full.
- The field blank will likely be dry due to its minimal exposure to the atmosphere (unless sampling is done on a rainy day).

Digestion Process:

1. Fill a 600 mL glass beaker around 1/3rd of the way full with ice.
2. Go to a fume hood.
3. Use a graduated cylinder to measure 10 mL of 30% hydrogen peroxide.
4. Pour that 10 mL of 30% hydrogen peroxide into a 10 mL glass beaker.
5. Remove the tinfoil from one of your beakers with a filter inside.
6. Lift the filter over a clean, 150 mL glass beaker using tweezers.
7. Use a glass pipette to gently, and fully, rinse the filter, allowing the solution to fall into the 150 mL glass beaker.
    a. Rinse the filter as thoroughly as possible.
8. Use a stainless steel spatula to gently scrape off any excess visible particles still stuck to the filter.
    a. Do this step very gently so as not to tear/rip the filter.
    b. Be slow and thorough to ensure you have removed all particles from the filter as best as possible.
9. Cover the beaker containing your hydrogen peroxide solution with tinfoil.
10. Use a temperature gun to measure the temperature of the solution inside the beaker for 10 minutes.
    a. If the temperature exceeds 50°C, place the beaker inside the 600 mL glass beaker with ice.
11. Place the covered beaker containing the hydrogen peroxide solution into an oven.
12. Bake the sample for 5-7 days at 55°C to ensure that the hydrogen peroxide is completely dissolved from the sample.
13. After the hydrogen peroxide has fully dissolved, remove the beaker from the oven.

Finalizing Filters:

1. Place a Büchner funnel rubber stopper on top of a filtering flask.
2. Place a 25 mm Büchner funnel on top of the rubber stopper.
3. Use a vacuum tube to connect the filtering flask to a vacuum source.
4. Place a 25 mm **PCTE**** filter on top of the Büchner funnel setup.
5. Remove the tinfoil covering the beaker.
6. Rinse the walls of the beaker with Milli-Q water.
7. Turn on the vacuum.
8. Slowly pour the contents from the beaker into the filtration setup.
    a. Pour a little bit incrementally until the beaker is empty.
    b. Turn off the vacuum.
9. Use a stainless steel spatula to scrape off any visible debris within the beaker.
10. Repeat steps 6-9.
11. Rinse the beaker with 96% ethanol.
12. Repeat steps 7-9.
13. Rinse the beaker again with 96% ethanol.
14. Repeat steps 7-8.
15. Use stainless steel tweezers to slowly and gently place the filter onto a clean, glass microscope slide.
16. Gently secure the edges of the filter in place using electrical tape.
    a. Make sure that the filter is just barely covered by tape and only enough to secure in place.
    b. Keep filter as flat as possible.
17. Gently place the microscope slide with the filter secured onto it into a clean, glass petri dish.

****RECOMMENDATION:** We recommend using a non-polymeric filter instead of the PCTE filter, which would allow for in-situ micro-Raman spectroscopy on the filter.

Visual Identification:

1. Gently remove microscope slide from petri dish.
2. Scan using an EVOS FL Auto fluorescence microscope.
    a. Use settings of 10x magnification and Brightfield.
3. Place microscope slide back into petri dish.
4. Open the scan of the filter in the ImageJ software.
5. Use ImageJ to overlay 6 evenly-sized rectangles that represent 50% of the total filter area.
    a. Each rectangle should have dimensions of roughly 3.7 mm x 11.1 mm.
    b. Overlay these rectangles randomly.
6. Use the ImageJ "add points" feature to identify potential MPs within your 6 rectangles.

    a. Follow criteria by Hidalgo-Ruz et al. (2012).
7. Double the count of potential MPs found within the rectangles to provide an estimate of the total potential MPs on each filter.

Micro-Raman Analysis:

1. Identify a subset of the total potential MPs on each filter.
    a. Roughly 5% was used for field samples
    b. Roughly 12.5% was used for field blanks
2. Place double-sided tape onto a microscope slide.
3. Place that microscope slide under LEICA S9E stereomicroscope.
4. Place microscope slide with finalized filter under LEICA S9E stereomicroscope.
5. Look under microscope to identify potential MPs following same criteria from Hidalgo-Ruz et al. (2012).
6. Using clean stainless steel tweezers, remove potential MPs from finalized filter onto the microscope slide with double-sided tape.
    a. After placing each MP onto the double-sided tape, circle each MP with a sharpie.
        i. Provide a wide enough berth around the MP to prevent getting any sharpie on the MP.
        ii. Keep track of color, shape, location of MP within sharpie circle, sampling period, sampling site, and MP ID (ex: #4).
7. Repeat steps 5&6 until the total subset of potential MPs has been isolated.
8. Place microscope slide with finalized filter back into its petri dish.
9. Move microscope slide with double-sided tape and isolated MPs to a HORIBA XploRA PLUS Confocal Raman Microscope with the following settings:
    a. 532 nm laser
    b. 600 gratings mm^{-1}
    c. 100-3500 cm^{-1} Raman shift range
    d. Laser filter of 10% or 25%
    e. Acquisition time = 4 seconds
    f. Accumulations = 4
    g. Slit size = 100 μm
    h. Hole = 300 μm
10. Use the HORIBA XploRA PLUS Confocal Raman Microscope to acquire each MP spectrum for 2 different locations on each MP.
11. Upload each MP spectrum into Open Specy using settings of:
    a. Threshold Signal-Noise:
        i. Minimum Value = 4
        ii. Signal Thresholding Technique = Signal Over Noise
    b. Min-Max Normalize checked
    c. Smoothing/Derivative:
        i. Polynomial = 3
        ii. Derivative Order = 1

iii. Wavenumber Window = 90
iv. Absolute Value checked
d. Conform Wavenumbers:
i. Conformation Technique = Linear Interpolation
ii. Wavenumber Resolution = 8
e. Intensity Adjustment:
i. Intensity Units = Absorbance
f. Baseline Correction:
i. Baseline Correction Polynomial = 8
g. Range Selection:
i. Minimum Wavenumber = 100
ii. Maximum Wavenumber = 3500
h. Flatten Region:
i. Minimum Wavenumber = 2200
ii. Maximum Wavenumber = 2400

12. If both recorded spectra for a MP were not definitive (yielding a Pearson's R value ≥ 0.7 for a plastic material), a third spectrum was acquired.
    a. If the third spectrum for a potential MP was not definitive, the particle was determined to not be plastic.
13. Potential MPs were confirmed as MPs by having a Pearson's R value ≥ 0.7 for a plastic material.

Notes:

- Make sure that HORIBA XploRA PLUS Confocal Raman Microscope has been properly calibrated recently using silicon.

Scaling Procedures:

1. Use the equation below to identify the amount of MPs on each field blank:

$$MP_{Total} = MP_{Total,p} * VP$$

Where,

MP_{Total} = Estimated total MP counts on each filter

$MP_{Total,p}$ = Total potential MPs on each filter from visual identification

VP = Verified proportion of MPs from micro-Raman spectroscopy (ex: 5 confirmed/10 in the subset = 5/10)

2. Average the MP_{Total} for each all field blanks.
3. Use the below equation to identify the amount of MPs on each field sample:

$$MP_{Total} = MP_{Total,p} * VP - \varepsilon$$

Where,

ε = Sampling error (Average MPs from field blanks)

4. Find the relative deposition rate for each field sample using the below equation:

$$Relative\ Deposition\ Rate = \frac{MP_{Total}}{(Area\ of\ field\ sampler\ [m^2])(Duration\ of\ field\ sampling\ [days])}$$

<u>Notes:</u>

- The area of the field sampler used in this study was approximately 0.043 m^2.
- The duration of field sampling for this study was 21 days.

References

[1] Montgomery WeatherSTEM, “Data mining,” weatherstem.com. https://montgomery.weatherstem.com/data?refer=/vt

[2] S. L. Wright, J. Ulke, A. Font, K. L. A. Chan, and F. J. Kelly, “Atmospheric microplastic deposition in an urban environment and an evaluation of transport,” Environment International, vol. 136, p. 105411, Mar. 2020, doi: 10.1016/j.envint.2019.105411.

[3] C. Liu *et al.*, “Widespread distribution of PET and PC microplastics in dust in urban China and their estimated human exposure,” *Environment International*, vol. 128, pp. 116–124, Jul. 2019, doi: 10.1016/j.envint.2019.04.024.

[4] R. Akhbarizadeh, S. Dobaradaran, M. Amouei Torkmahalleh, R. Saeedi, R. Aibaghi, and F. Faraji Ghasemi, “Suspended fine particulate matter (PM2.5), microplastics (MPs), and polycyclic aromatic hydrocarbons (PAHs) in air: Their possible relationships and health implications,” *Environmental Research*, vol. 192, p. 110339, Jan. 2021, doi: 10.1016/j.envres.2020.110339.

[5] S. Allen et al., “Atmospheric transport and deposition of microplastics in a remote mountain catchment,” Nat. Geosci., vol. 12, no. 5, pp. 339–344, May 2019, doi: 10.1038/s41561-019-0335-5.

[6] E. Knobloch, H. Ruffell, A. Aves, O. Pantos, S. Gaw, and L. E. Revell, “Comparison of Deposition Sampling Methods to Collect Airborne Microplastics in Christchurch, New Zealand,” *Water Air Soil Pollut*, vol. 232, no. 4, p. 133, Apr. 2021, doi: 10.1007/s11270-021-05080-9.

[7] K. Liu, X. Wang, T. Fang, P. Xu, L. Zhu, and D. Li, “Source and potential risk assessment of suspended atmospheric microplastics in Shanghai,” *Science of The Total Environment*, vol. 675, pp. 462–471, Jul. 2019, doi: 10.1016/j.scitotenv.2019.04.110.

[8] X. Wang, C. Li, K. Liu, L. Zhu, Z. Song, and D. Li, “Atmospheric microplastic over the South China Sea and East Indian Ocean: abundance, distribution and source,” *Journal of Hazardous Materials*, vol. 389, p. 121846, May 2020, doi: 10.1016/j.jhazmat.2019.121846.

[9] J. Brahney, M. Hallerud, E. Heim, M. Hahnenberger, and S. Sukumaran, “Plastic rain in protected areas of the United States,” Science, vol. 368, no. 6496, pp. 1257–1260, Jun. 2020, doi: 10.1126/science.aaz5819.

[10] S. Abbasi and A. Turner, “Dry and wet deposition of microplastics in a semi-arid region (Shiraz, Iran),” *Science of The Total Environment*, vol. 786, p. 147358, Sep. 2021, doi: 10.1016/j.scitotenv.2021.147358.
